# CuFe_2_O_4_@SiO_2_@L-arginine@Cu(I) as a new magnetically retrievable heterogeneous nanocatalyst with high efficiency for 1,4-disubstituted 1,2,3-triazoles synthesis

**DOI:** 10.1038/s41598-023-36012-8

**Published:** 2023-05-29

**Authors:** Fatemeh Salehzadeh, Maryam Esmkhani, Mahsa Zallaghi, Shahrzad Javanshir, Mohammad G. Dekamin

**Affiliations:** Pharmaceutical and Heterocyclic Compounds Research Laboratory, Department of Chemistry, Iran University of Science and Technology, Tehran, 16846-13114 Iran

**Keywords:** Chemistry, Materials science

## Abstract

A novel magnetic heterogeneous catalyst was synthesized through the immobilization of copper ions onto the l-arginine functionalized CuFe_2_O_4_@SiO_2_. The prepared catalyst was characterized by Fourier Transform Infrared (FT-IR), X-ray diffraction (XRD), Field emission scanning electron microscopy (FE-SEM), Transmission electron microscopy (TEM), and Energy Dispersive X-Ray spectroscopy (EDX). The resulting catalyst was used in the ultrasonic-assisted synthesis of 1,2,3-triazoles via a one-pot three-component reaction of alkynes, alkyl halides, and sodium azides under green conditions within a short time. The catalyst reusability was investigated after five cycles and no significant loss of activity was observed.

## Introduction

Minimizing the generation of hazardous substances is an important obligation of green chemistry. Green catalytic processes, which include the use of non-toxic solvents (water, EtOH, etc.), reusable, efficient catalysts, and new synthetic route can pleasantly follow the principles of green chemistry^1–3^. Recently, sonochemistry has turned into an attractive synthetic technique in a green manner^4^. It contains many features, such as reducing hazardous chemicals, solvents, and energy consumption. The ultrasound mechanism is related to spontaneous creation, growth, and collapse of bubbles formed during the acoustic cavitation process, which can accelerate the reaction rate^5^. The release of a considerable amount of heat prepares the required energy for the reaction to driving forward. These unusual properties justify its widely uses in the synthesis of organic and inorganic materials^6^.

Triazole systems as important five-membered ring structures composed of three nitrogen atoms are found in many pharmaceutical and agrochemical structures. They possess wide biological activities such as: anti-inflammatory^7^, antimicrobial^8^, antimalarial^9^, antiviral^10^, and anticancer^11^ activities. These versatile scaffolds have been specified in numerous clinically used drugs emphasizing their importance. Due to the importance of these heterocyclic structures, they can be readily synthesized in click reaction. The Huisgen reaction is the first example of to click reaction in which the 1,3-dipolar cycloaddition of azides to alkynes catalyzes by copper and forms the five-membered heterocycles^12^. This concerted [3 + 2] thermally cycloaddition cannot be proceeded in the absence of catalyst. Metal-based catalysts were employed over the years to defeat this deficiency^13,14^.

The homogeneous catalytic systems like copper nanoparticles^15^, copper nanoclusterss^16^, and in-situ reduction of Cu(II) salts to Cu(I) salts^17^, have some disadvantages related to the recovering and reusing ability for successive reaction cycles and the presence of metal contamination in the end product. The utilization of heterogeneous catalysts can be a promising solution overcoming this problem. The heterogeneous catalysts should compete with each other in economical and environmentally friendly subjects. There have been many surfaces reported for copper immobilizing so far, such as: CuO hollow nanosphere^18^, shillajit^19^, charcoal^20^, SBA-15^21^ and so on which often suffer from separating and leakage problems. So, using magnetic supporters can be a good choice to attain easy separation, thermal stability, and low toxicity properties.

But the major disadvantages associated with these homogeneous copper catalysts are the difficulties to recover and reuse for successive reaction cycles and the possibility of metal contamination with the end product. To overcome these serious issues, various solid-supports like zeolites [53], polymers [54,55], carbon [44], silica [56] etc. have been employed to synthesize the corresponding heterogeneous copper catalysts by immobilizing the active metal ions onto the solid supports.

**Figure 1:**
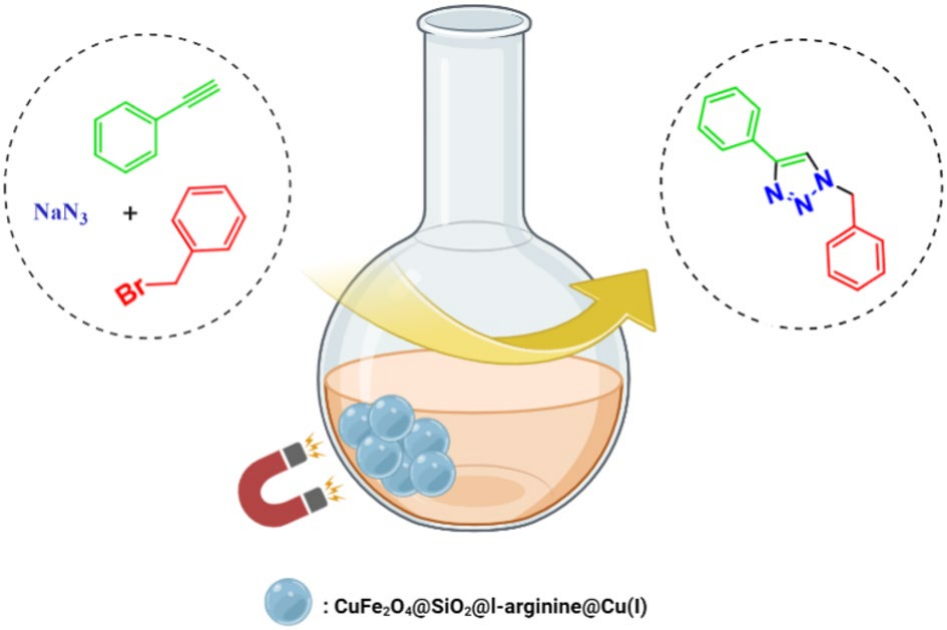
CuFe_2_O_4_@SiO_2_@l-arginine@Cu (I) as an appropriate catalyst for click reaction. (Created with BioRender.com).

In continuation of our work on the synthesis of heterocyclic structures^22–24^, we reported the synthesis of a new efficient magnetite-base catalytic system, CuFe_2_O_4_@SiO_2_@l-arginine@Cu, along with its application in an approach to 1,2,3-triazole derivatives. The procedure uses phenylacetylene as an alkyne, sodium azide, and various alkyl halides as the other component to form triazoles. The reaction was done in ultrasonic-assisted green conditions and the catalyst removed with an external magnet (Fig. 1). The reaction yields were excellent and the prepared catalyst has a good efficiency even after five cycles.

## Experimental section

### Materials

All the reactants were purchased from Merck Chemical Company and Aldrich and used without further purification. Fourier transform infrared (FT-IR) spectra were recorded as KBr pellets using a Bruker VRTEX 70 model FT-IR spectrophotometer. Powder X-ray diffraction (XRD) patterns were collected with a Rigaku-Dmax 2500 diffractometer with nickel filtered Cu Kα radiation (*λ* = 1.5418 Å, 40 kV). Supermagnetic properties of the catalyst were measured with a vibrating sample magnetometer at room temperature.

### Preparation of CuFe2O4@SiO2 nanoparticles

CuFe_2_O_4_ was readily synthesized using a chemical co-precipitation method previously reported^25^, followed by a SiO_2_-coating procedure^26^. Briefly, 2.00 g of the obtained CuFe_2_O_4_ was dispersed in a mixture of 100 mL of ethanol, 40 mL of deionized water and 6 mL of concentrated aqueous ammonia solution, followed by the addition of 4 mL tetraethylorthosilicate (TEOS). This solution was stirred mechanically at room temperature overnight. Then the product, CuFe_2_O_4_@SiO_2_, was separated using an external magnet, washed with deionized water and ethanol three times, and dried at room temperature.

### Preparation of CuFe2O4@SiO2@l-arginine@Cu(I)

In the second step, CuFe_2_O_4_@SiO_2_@l-arginine nanocatalyst was synthesized using the following procedure. An amount of 1 g of CuFe_2_O_4_@SiO_2_ was suspended in deionized water (20 mL) and became highly dispersed via sonication. Then, 2 g of l-arginine was added and the mixture was stirred at 90 °C for 15 h. CuFe_2_O_4_@SiO_2_@l-arginine nanoparticles were separated from the aqueous solution by applying an external magnet, washed with distilled water and then dried in an oven. The whole synthesis was done under an inert atmosphere. In the last step, incorporation of copper onto the CuFe_2_O_4_@SiO_2_@l-arginine nanocomposite was carried out by mixing the CuFe_2_O_4_@SiO_2_@l-arginine (1 g) and CuI (0.5 g) in absolute ethanol (50 mL). The mixture was refluxed for 24 h. Cu(I) ions were adsorbed onto the magnetic nanocarrier. Finally, the synthesized CuFe_2_O_4_@SiO_2_@l-arginine@Cu(I) nanocomposite as a brown powder was separated from the suspension using magnetic decantation, washed with absolute ethanol and dried under vacuum at room temperature.

### General procedure for preparation of triazoles

A mixture of CuFe_2_O_4_@SiO_2_@l-arginine@Cu(I) (1 mol% of Cu), benzyl halide (1.0 mmol), phenylacetylene derivatives (1.2 mmol), and NaN_3_ (1.2 mmol) in a 1:1 mixture of H_2_O:EtOH (3 ml) was irradiated under sonication for an appropriate time (Tables S1 and S2). After completion of the reaction monitored by TLC, the catalyst was separated with an external magnet and the solvents were removed under vacuum evaporator and the product was further purified by EtOH/water system.

## Results and discussion

**Figure 2:**
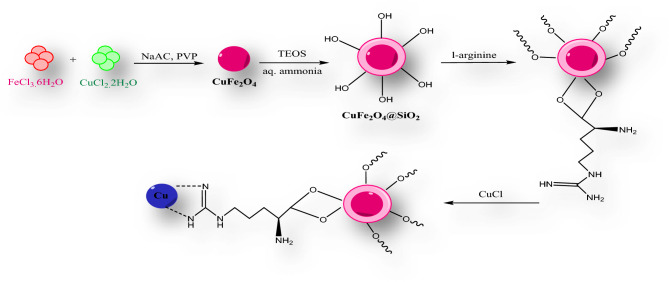
Schematic representation of the catalyst synthesis procedures.

The synthetic pathway of CuFe_2_O_4_@SiO_2_@l-arginine@Cu(I) is illustrated in Fig. 2. CuFe_2_O_4_ NPs were prepared through a co-precipitation method by dissolving salts into distilled water, followed by precipitation with NH_4_OH. Afterward, TEOS was hydrolyzed to form silica oligomers, which were coated on the surface of CuFe_2_O_4_ nanoparticles to obtain CuFe_2_O_4_@SiO_2_ nanoparticles. CuFe_2_O_4_@SiO2@l-arginine was obtained by nucleophilic addition of arginine to as-prepared magnetic nanoparticles. Subsequently, the copper was linked to the nitrogen groups of arginine.

### Characterization of the CuFe2O4@SiO2@l-arginine@Cu(I)

The structure, morphology and magnetic properties of the prepared catalyst were entirely characterized by analytical techniques. The FT-IR spectra of the CuFe_2_O_4_@SiO_2_@l-arginine@Cu, CuFe_2_O_4_@SiO_2_, and CuFe_2_O_4_ are compared in Fig. S1 (see supporting information). The FTIR spectrum of CuFe_2_O_4_ indicates the characteristic bands of metal–O at 645 cm^−1^, 579 cm^−1^ and 479 cm^−127^ and the absorption peak at 3430 cm^−1^ is ascribed to the OH stretching vibration, indicating the presence of hydrogen bonded hydroxyl groups on the surface of CuFe_2_O_4_ nanoparticles^35^ . The sharp bands at 1072 cm^−1^ and 816 cm^−1^ were assigned to the asymmetric and symmetric stretching vibrations of Si–O–Si bonding respectively. In Fig. S1c, the absorption peak at 3426 cm^−1^ was assigned to the O–H stretching vibrations which shifted from 3426 to 3276 cm^-1^ in CuFe_2_O_4_@SiO_2_@l-arginine with a net reduction in the intensity indicating the involvement of arginine in the synthesis of composite. Moreover, C=N stretching vibrations in the FTIR spectrum of the final catalyst appear at 1629 cm^−1^ which is lower than C=N stretching vibrations in the FTIR spectrum of CuFe_2_O_4_@SiO_2_@l-Arginine due to the formation of metal-ligand bonds.

**Figure 3:**
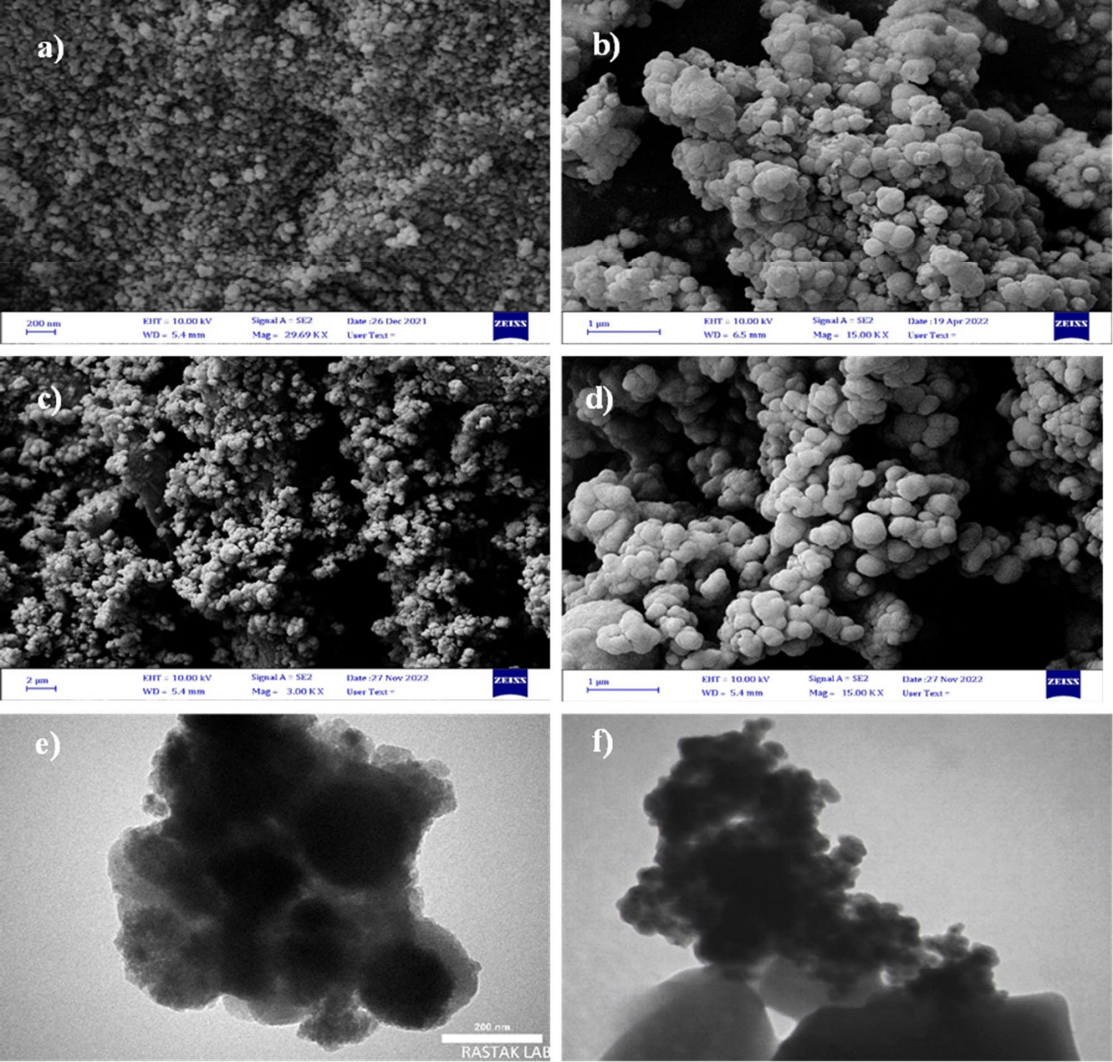
SEM images of (**a**, **b**) CuFe_2_O_4_@SiO_2_@l-arginine-Cu (I), (**c**, **d**) Reused catalyst after 4 runs, and (**e**, **f**) TEM images of CuFe_2_O_4_@SiO_2_@l-arginine-Cu (I).

The morphology and the structure of the CuFe_2_O_4_@SiO_2_@l-arginine@Cu(I) was characterized by SEM and TEM analysis (Fig. 3a–f). The almost uniform distribution and spherical structure of the catalyst is clearly observable in SEM images. The core-shell structure of the magnetic particles was proofed via TEM analysis with the black centers and the brightest areas as CuFe_2_O_4_ cores and SiO_2_ shells, respectively.

The Brunauer–Emmett–Teller (BET) method was applied to calculate the surface area and pore diameter of the prepared catalyst. According to the BET analysis results presented in Fig. S2 (See supporting information), the surface area and average pore diameter are 33.65 m^2^/g and 17.59 nm for CuFe_2_O_4_@SiO_2_@L-arginine@Cu(I) catalyst.

To determine the oxidation states of Cu in the prepared catalyst, XPS analysis was performed. The XPS analysis of the CuFe_2_O_4_@SiO_2_@L-arginine@Cu(I) nanoparticles (Fig. S3, supporting information) revealed the characteristics peaks for C 1 s (284.88), O 1 s (530.39), Fe 2p (710.89) and Cu 2p (933.01). The Cu2p3/2 peaks located at 933.0 eV was attribute to Cu^1^.

**Figure 4:**
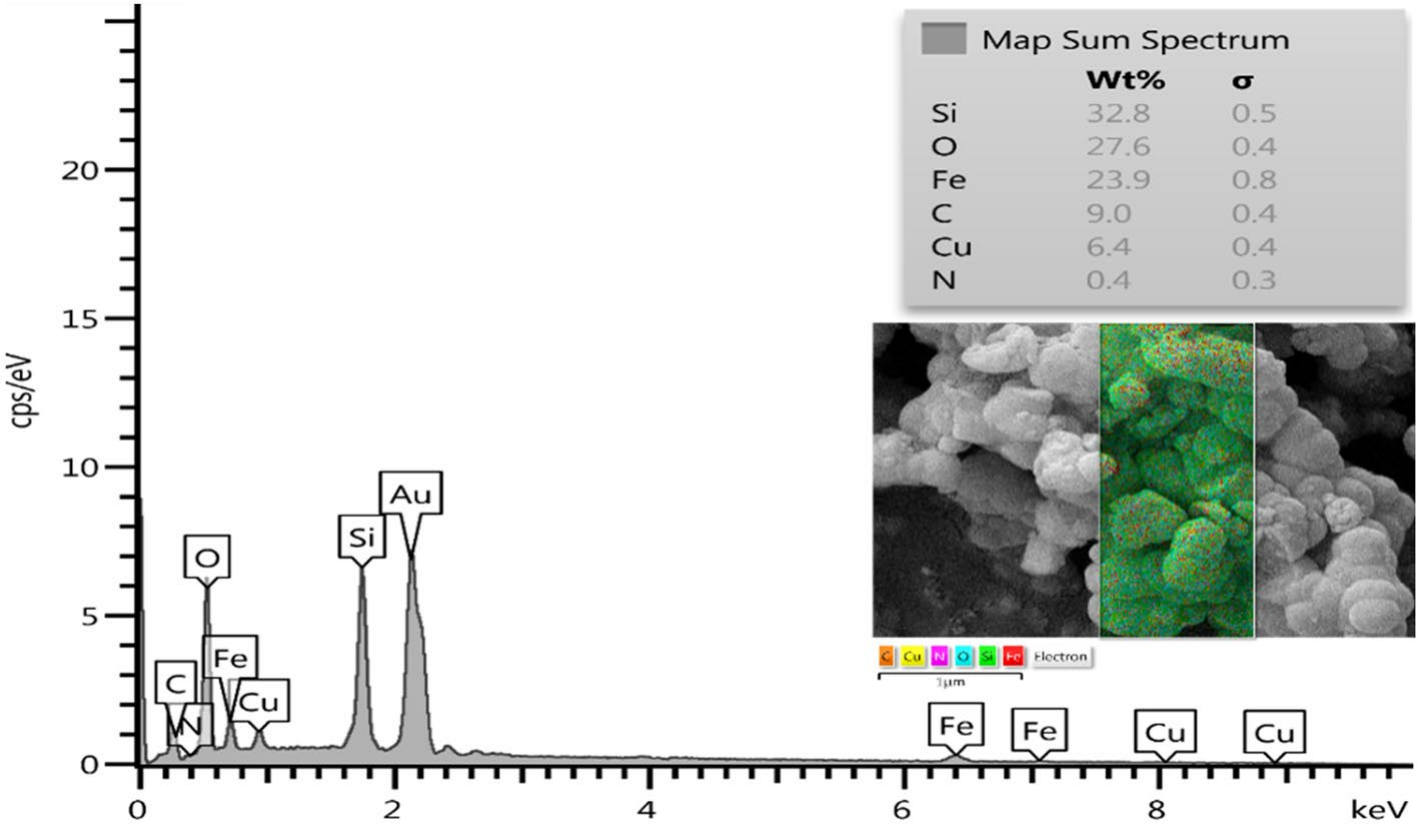
Energy dispersive X-ray analysis (EDX) of CuFe_2_O_4_@SiO_2_@l-arginine@Cu (I) and b) elemental mapping of C (orange); N (purple), O (blue), Fe (red), Si (green), and Cu (yellow) atoms for CuFe_2_O_4_@SiO_2_@l-arginine@Cu (I).

The EDS analysis results confirmed the presence of carbon, oxygen, nitrogen, copper, iron, and Si elements in the catalyst (ratios of 9.0: 27.6: 0.4: 6.4: 23.9: 32.8 wt%, respectively) shown in Fig. 4 and inset. It also confirms the immobilization of Cu on CuFe_2_O_4_@SiO_2_@l-arginine was achieved successfully. Moreover, the accurate amount of copper in the final catalyst composition determined via ICP analysis was 9.14%.

The magnetic properties of CuFe_2_O_4_, CuFe_2_O_4_@SiO_2_ and CuFe_2_O_4_@SiO_2_@l-arginine@Cu (I) were studied using VSM analysis at ambient temperature with the magnetic field sweeping from − 10,000 to + 10,000 Oe, and the magnetization cycles are shown in Fig. S4 (see supporting information). Obviously, the particles showed zero remanent magnetization which is the reason for their superparamagnetic behavior. Superparamagnetic nanoparticles would not aggregate magnetically due to the lack of net magnetization in the absence of an external field^27,28^. Magnetic hysteresis loop measurements revealed the maximum saturation magnetization value of CuFe_2_O_4_@SiO_2_@l-arginine@Cu (I) was less than CuFe_2_O_4_ (58.2 emus. g^-1^) which proved the incorporation of arginine and copper on the surface of CuFe_2_O_4_.

The crystal structure of the prepared samples was examined using X-ray diffraction pattern (XRD) shown in Fig. S5 (See supporting information). the XRD pattern of CuFe_2_O_4_ revealed characteristic diffraction peaks at 2*θ* = 30.206°, 35.579°, 43.361°, 50.475, 53.678°, 57.146°, 62.788°, 74.296° corresponding to the (220), (311), (400), (107), (422), (511), (440), and (533) reflection crystal plans of CuFe_2_O_4_ respectively (JCPDS card no. 25-0283) which have been reported in the literature^29^. The slightly broad diffraction peak at 2*θ* value 20–30° was attributed to the amorphous silica indicated the formation of SiO_2_ shell does not change the crystal form of CuFe_2_O_4_ (JCPDS card no.00-002-0278). The XRD pattern of CuFe_2_O_4_@SiO_2_@l-arginine@Cu(I) revealed a sharp peak at 28.438 attributed to CuI. Moreover, the XRD pattern of reused CuFe_2_O_4_@SiO_2_@l-arginine@Cu(I) showed that the crystalline structure of catalyst remained unchanged after several runs. (The reference card numbers were collected from the X'pert HighScore Plus version 1.0d software developed by the PANalytical B.V.)

Thermal behavior of the prepared catalyst was analyzed using TGA and DTG under Ar atmosphere at a temperature varying from 50 to 800 °C and the plotted curve shown in Fig. S6 (See supporting information). The TGA thermogram of CuFe_2_O_4_@SiO_2_@L-arginine@Cu(I) shows two stage weight loss over the temperature range of TG analysis. The first stage, including a low amount of weight loss (6%) at T ~ 110 °C, resulted from the release of both the physiosorbed and chemisorbed water, the second stage at about 290 °C to nearly 460 °C is attributed to the decomposition of the organic moiety in the nanocomposite including a weight loss (45%).

### Evaluation the catalytic activities of CuFe_2_O_4_@SiO_2_@l-arginine-Cu(I) in the synthesis of 1,2,3-triazole derivatives

The catalytic behavior of CuFe_2_O_4_@SiO_2_@l-arginine-Cu(I) was investigated for the synthesis of triazole derivatives via a three-component reaction between sodium azide, phenylacetylene, and benzyl halide under different conditions. To find the optimal reaction conditions, various factors such as catalyst loading, solvent, time and reaction temperature were scrutinized in a model reaction including phenylacetylene, benzyl bromide, and sodium azide presented in Table S1 (see supporting information).

For further optimization, the type of catalyst was also investigated and tabulated in Table S2 (See supporting information). The results revealed the high performance of CuFe_2_O_4_@SiO_2_@l-arginine-Cu(I) due to synergistic effects and improved number of active sites on the surface. The conversion of 87% was reached for 15 mg catalyst loading under ultrasonic irradiation. Obviously, the increase in catalyst loading was not favorable. On the other hand, with an amount of catalyst of 30 mg, the yield did not change significantly compared to 15 mg.

**Figure 5:**
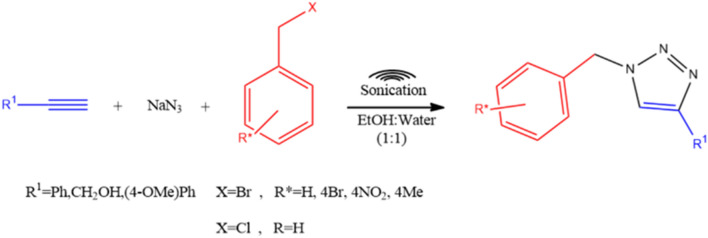
Schematic representation of the CuFe_2_O_4_@SiO_2_@l-arginine@Cu(I) in one-pot synthesis of 1,2,3 triazoles (4a-l).

To generalize the optimum conditions, different 1,2,3-triazole derivatives from 4a–j were prepared through a one-pot reaction of acetylene derivatives 1, sodium azide 2 and benzyl halide derivatives in the presence of CuFe_2_O_4_@SiO_2_@l-arginine@Cu(I) (Fig. 5). The results are summarized in Table 1. As expected, the presence of electron-withdrawing groups on benzyl halide can enhance the rate and yield of the reaction. On the other hand, the reaction with benzyl bromide is much better than benzyl chloride. It can perhaps be because of the fact that -Br is a good leaving group in the substation reaction of azide anion.

**Table 1:** Synthesis of 1,2,3 triazoles in the presence of CuFe_2_O_4_@SiO_2_@l-arginine@Cu(I).

Entry	R_1_	R*	X	Time (min)	TON	TOF (S^-1^)	m.p. °C (reported)	Product
1	Ph	H	Cl	25	4761	3.17	129–130 (128–130)^31^	4a
2	Ph	H	Br	20	4867	4.05	129–130 (129–130)^23^	4b
3	Ph	2-Cl	Cl	20	4656	3.88	84–85 (84–86)^19^	4c
4	Ph	4-NO_2_	Br	30	2804	1.56	140–141 (140–141)^32^	4d
5	Ph	4-Me	Br	20	4761	3.97	91–92 (91–92)^33^	4e
6	Ph	4-Br	Br	15	5026	5.6	150–152 (150–152)^32,34^	4f
7	Ph	4-OMe	Cl	25	3915	2.6	135 (133–135)^35^	4 g
8	(4-OMe)Ph	2-Cl	Cl	20	4338	3.6	128 (129–134)^36^	4 h
9	(4-OMe)Ph	4Br	Cl	15	4761	5.3	165–167 (164–166)^19^	4i
10	–CH_2_OH	H	Br	15	2010	2.23	78–79 (78–80)^37^	4j
11	–CH_2_OH	H	Cl	20	1904	1.6	78–79 (78–79)^38^	4 k
12	–CH_2_OH	4-Br	Br	20	2380	2.0	117–119 (117–121)^34^	4 l

*Reaction condition: Acetylene (1 mmol), Sodium azide (1.1 mmol), benzyl halide (1 mmol), 15 mg catalyst, and 3 ml solvent.

In addition, for better characterization of the products the ^1^HNMR spectra of the samples 4 h and 4i have been represented in Figs. S8 and S9 respectively (see supporting information).

Additionally, the efficiency of the catalyst was shown via the turnover number (TON) and turnover frequency (TOF) of the catalyst and provided in Table 1. As can be seen, the obtained values of TOF are between 2 and 5.6 S^-1^, which is very valid for relevant industrial applications, for which the TOF is in the range10^−2^ and 10^2^ S^−1^^30^.

**Figure 6:**
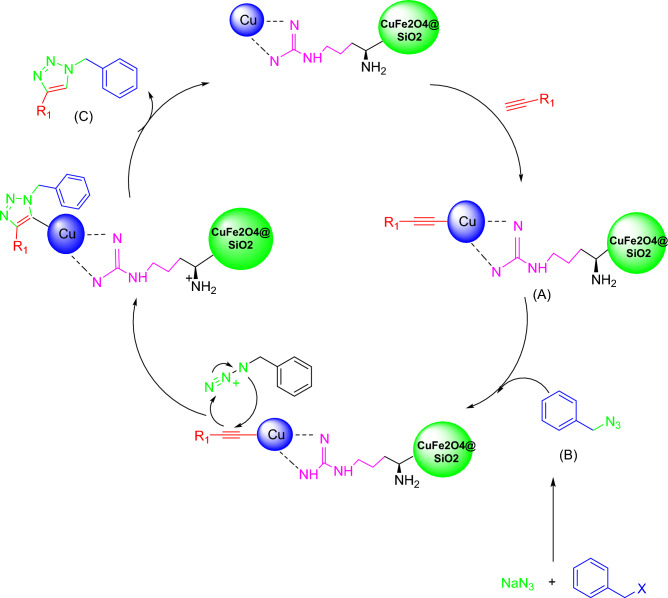
Proposed catalytic mechanism of CuFe_2_O_4_@SiO_2_@l-arginine@Cu(I).

The proposed mechanism of the model reaction for triazole derivatives synthesis is mentioned in Fig. 6. In the first step the bifunctional catalyst forms copper acetylide (A). On the other hand, the organic azide was synthesized in-situ by the reaction of aryl halide with NaN_3_. Then the coordination of the organic azide to the copper acetylide was occurred and by the Huisgen 1, 3-dipolar cycloaddition reaction of (A) and (B) the final desired 1,2,3 triazole (C) obtained.

### Hot filtration

The hot filtration test was carried out to investigate the heterogeneous nature of the CuFe_2_O_4_@SiO_2_@l-arginine@Cu(I) in the synthesis of 1,2,3 triazole. At first, the model reaction was performed under the optimized reaction condition. After 10 min (43% conversion), the catalyst was removed from the reaction by an external magnet and also simple filtration. The reaction was then allowed to proceed without catalyst for 30 min. The results showed that the reaction did not progress in the absence of CuFe_2_O_4_@SiO_2_@l-arginine@Cu(I), thus proving the heterogeneity of the catalyst and the non-leaching of copper in the solution.

### Catalyst recyclability

The easy separation of CuFe_2_O_4_@SiO_2_@l-arginine@Cu(I) as a heterogeneous catalyst was mentioned before. In this regard, the recyclability of the nanocatalyst in the model reaction was investigated. At the end of the reaction, CuFe_2_O_4_@SiO_2_@l-arginine@Cu(I) was collected by an external magnetic field and washed with ethanol and water. The dried magnetic nanocatalyst was successively used for five times in the model reaction with a yield of 75%. According to the results displayed in Fig. S7 (see supporting information), there is no significant reduction in the catalytic efficiency of CuFe_2_O_4_@SiO_2_@l-arginine@Cu(I). Furthermore, according to the FESEM images shown in Fig. 3c,d there is no structural changes in catalyst after 5 times recycling. FTIR spectra of the fresh and recycled catalyst were shown in Fig. S1 (see Supporting Information). It is clear that the used catalyst has not undergone any structural changes.

In order to determine the catalytic efficacy of the prepared CuFe_2_O_4_@SiO_2_@l-arginine@Cu(I) in the preparation of 1,2,3 triazoles, the present work was compared with the previous reports. As it is obvious, the prepared catalyst has several advantages in the time of reaction time, solvent, and yield which are presented in Table 2.

**Table 2:** Comparison of the present catalyst for the synthesis of triazoles with reported studies.

Entry	Catalyst	Conditions	Time(min)	Yield (%)	References
1	Cu(I)‐AMPS	Water/R.T	60	82	^39^
2	Fe3O4@LDH@cysteine–Cu(I)	Choline azide/70 °C	25	90	^40^
3	Fe_3_O_4_@SiO_2_-pAMBA-CS-Cu_2_O	Water/70 °C	20	93	^23^
4	Cu_2_O@Peanut shell	EtOH:Water/50 °C	90	93	^41^
5	CuFe_2_O_4_@SiO_2_@l-arginine@Cu(I)	EtOH:Water/60 °C	35	89	This work

## Conclusions

In summary, we devised a novel collagen-coated superparamagnetic organic-inorganic hybrid catalyst, CuFe_2_O_4_@SiO_2_@l-arginine@Cu(I), which exhibited radically enhanced catalytic activity in the synthesis of a wide range of substituted 1,2,3 triazole derivatives through a one-pot atom economical Huisgen 1, 3-dipolar cycloaddition reaction of acetylene derivatives, sodium azide, and benzyl halide under ultrasonic irradiation. This heterogeneous catalyst efficiency is achieved in several aspects, such as high product yields and reactivity in a green manner, stability, recyclability, and high reaction rate. Furthermore, the easy separation and removal from the reaction make this catalyst a good choice for use in other synthetic applications. These results affirmed that the novel CuFe_2_O_4_@SiO_2_@l-arginine@Cu(I) can be used as a versatile catalyst for promoting chemical reactions.

## Supplementary Information

Supplementary Information.

## Data Availability

All data generated or analysed during this study are included in this published article [and its supplementary information files].
