# Supplementary material for: CuFe2O4@SiO2@L-arginine@Cu(I) as a new magnetically retrievable heterogeneous nanocatalyst with high efficiency for 1,4-disubstituted 1,2,3-triazoles synthesis
Source: Sci Rep. 2023 May 29;13:8675. doi: 10.1038/s41598-023-36012-8 (PMC10227041; doi:10.1038/s41598-023-36012-8)
Supplement: Supplementary file 1 — Supplementary Information. [file 41598_2023_36012_MOESM1_ESM.docx]

**Supporting Information**

**CuFe_2_O_4_@SiO_2_@l-arginine@Cu(I) as a new magnetically retrievable heterogeneous nanocatalyst with high efficiency for triazole synthesis**

Fatemeh Salehzadeh^1^, Maryam Esmkhani^1^, Mahsa Zallaghi^1^, Shahrzad Javanshir^1*^, Mohammad G. Dekamin^1^

^1^ Pharmaceutical and Heterocyclic Compounds Research Laboratory, Department of Chemistry, Iran University of Science and Technology, Tehran, 16846-13114, Iran

[**Figure S 1.** FT-IR spectra of the CuFe_2_O_4_, CuFe_2_O_4_@SiO_2_, CuFe_2_O_4_@SiO_2_@arginine@Cu(I) and Reused CuFe_2_O_4_@SiO_2_@arginine@Cu(I). 2](#_Toc123318964)

[**Figure S 2.** BET analysis of CuFe_2_O_4_@SiO_2_@arginine@Cu(I). 3](#_Toc123318965)

[**Figure S 3.** XPS analysis of CuFe_2_O_4_@SiO_2_@arginine@Cu(I). 3](#_Toc123318966)

[**Figure S 4.** VSM analysis of CuFe2O4, CuFe2O4@SiO2 and CuFe2O4@SiO2@l-arginine@Cu (I). 4](#_Toc123318967)

[**Figure S 5.** XRD pattern of: a) CuFe_2_O_4_, b) CuFe_2_O_4_@SiO_2_, c) CuFe_2_O_4_@SiO_2_@l-arginine@Cu (I). 4](#_Toc123318968)

[**Figure S 6.** TGA analysis of CuFe_2_O_4_@SiO_2_@l-arginine@Cu (I). 5](#_Toc123318969)

[**Figure S 7.** Recyclability of CuFe_2_O_4_@SiO_2_@l-arginine@Cu(I). 5](#_Toc123318970)

[**Table S 1.** Investigation of the prepared catalyst in different conditions in model reaction. 6](#_Toc123318971)

[**Table S 2.** Optimization of the type of catalyst. 6](#_Toc123318972)

[**Figure S 8.** ^1^HNMR spectra of the product 4h 7](#_Toc123318973)

[**Figure S 9.** ^1^HNMR spectra of the product 4i 8](#_Toc123318974)

**Figure S 1.** FT-IR spectra of the CuFe_2_O_4_, CuFe_2_O_4_@SiO_2_, CuFe_2_O_4_@SiO_2_@arginine@Cu(I) and Reused CuFe_2_O_4_@SiO_2_@arginine@Cu(I).


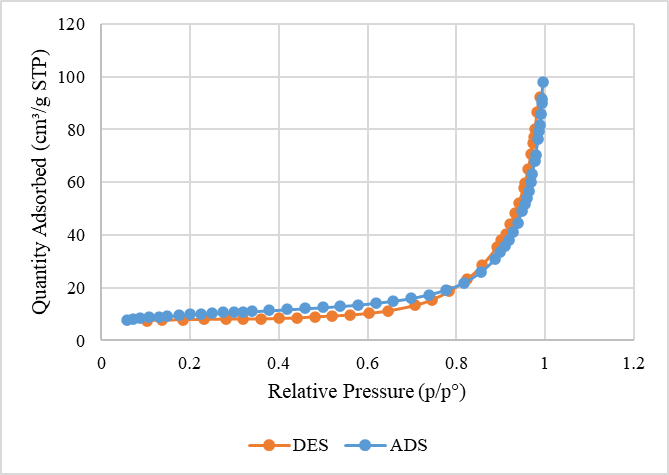


**Figure S 2.** BET analysis of CuFe_2_O_4_@SiO_2_@arginine@Cu(I).


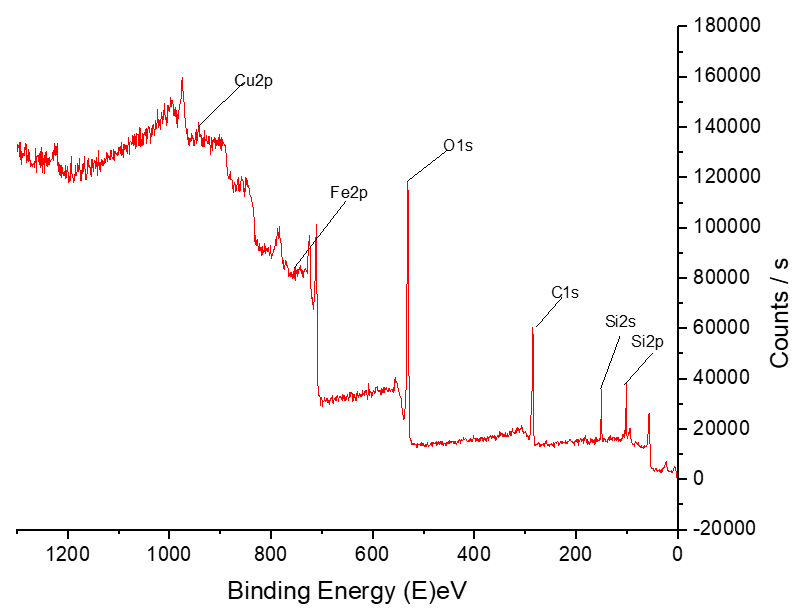


**Figure S 3.** XPS analysis of CuFe_2_O_4_@SiO_2_@arginine@Cu(I).

**Figure S 4.** VSM analysis of CuFe2O4, CuFe2O4@SiO2 and CuFe2O4@SiO2@l-arginine@Cu (I).


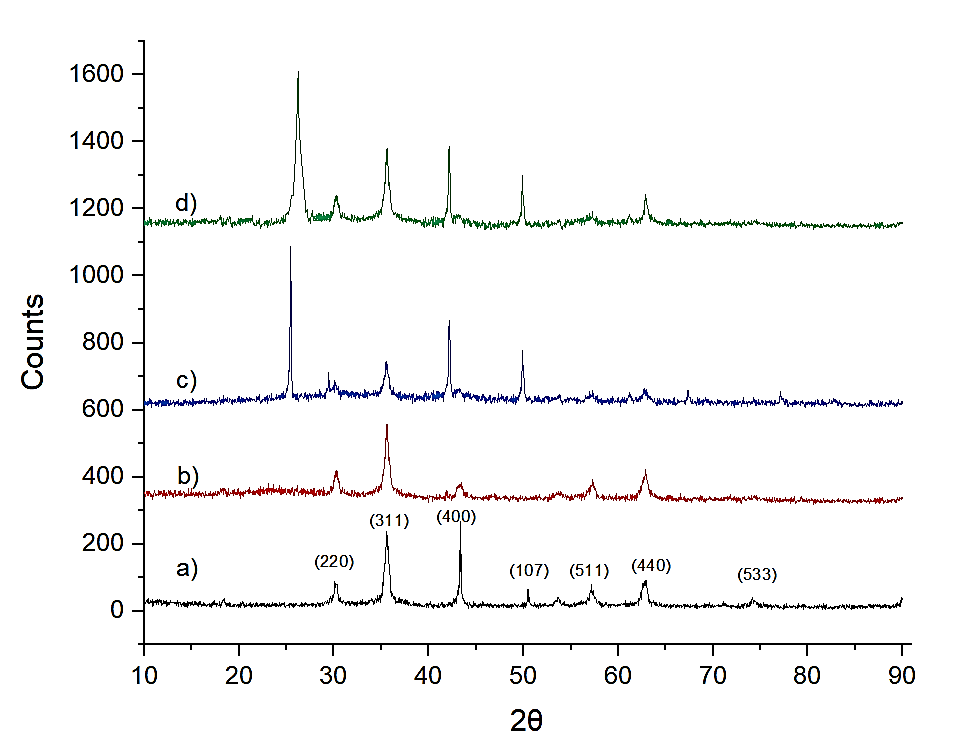


**Figure S 5.** XRD pattern of: a) CuFe_2_O_4_, b) CuFe_2_O_4_@SiO_2_, c) CuFe_2_O_4_@SiO_2_@l-arginine@Cu (I), and reused CuFe_2_O_4_@SiO_2_@l-arginine@Cu (I).


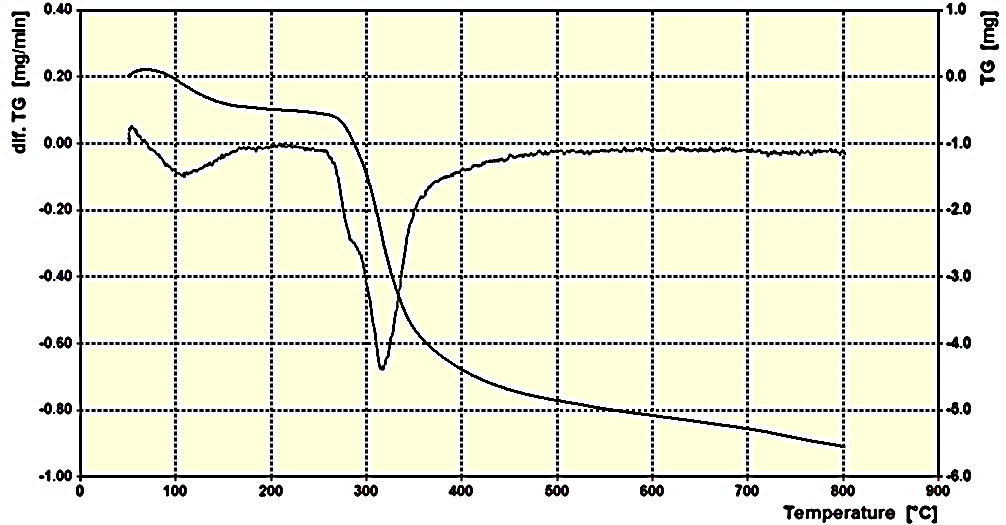


**Figure S 6.** TGA analysis of CuFe_2_O_4_@SiO_2_@l-arginine@Cu (I).

**Figure S 7.** Recyclability of CuFe_2_O_4_@SiO_2_@l-arginine@Cu(I).

**Table S 1.** Investigation of the prepared catalyst in different conditions in model reaction.

| Entry | Solvent | Catalyst^a^ (mol%) | Condition | Time (min) | Yield^b^ % |
| --- | --- | --- | --- | --- | --- |
| 1 | H_2_O | 1.90 | Reflux | 45 | 52 |
| 2 | EtOH | 1.90 | Reflux | 45 | 42 |
| 3 | H_2_O:EtOH(1:1) | 1.90 | Reflux | 45 | 35 |
| 4 | Toluene | 1.90 | Reflux | 45 | Trace |
| 5 | H_2_O | 1.90 | Ultrasonic | 25 | 55 |
| 6 | EtOH | 1.90 | Ultrasonic | 25 | 52 |
| 7 | Chloroform | 1.90 | Ultrasonic | 25 | 40 |
| 8 | MeCN | 1.90 | Ultrasonic | 25 | 45 |
| 9 | H_2_O:EtOH(1:1) | 1.90 | Ultrasonic | 25 | 65 |
| 10 | H_2_O:EtOH(1:2) | 1.90 | Ultrasonic | 25 | 60 |
| 11 | H_2_O:EtOH(1:1) | - | Ultrasonic | 25 | - |
| 12 | H_2_O:EtOH(1:1) | 0.95 | Ultrasonic | 25 | 69 |
| 13 | H_2_O:EtOH(1:1) | 1.90 | Ultrasonic | 25 | 65 |
| 14 | H_2_O:EtOH(1:1) | 2.85 | Ultrasonic | 25 | 87 |
| 15 | H_2_O:EtOH(1:1) | 3.80 | Ultrasonic | 25 | 79 |
| 16 | H_2_O:EtOH(1:1) | 5.71 | Ultrasonic | 25 | 89 |

^a^ Based on Cu content.

^b^Isolated yield.

**Table S 2.** Optimization of the type of catalyst.

| Entry | Catalyst (2.85 mol%) | Time (min) | Yield % |
| --- | --- | --- | --- |
| 1 | CuFe_2_O_4_ | 25 | 73 |
| 2 | CuFe_2_O_4_@SiO_2_ | 25 | 65 |
| 3 | CuFe_2_O_4_@SiO_2_@l-arginine | 25 | 68 |
| 4 | CuFe_2_O_4_@SiO_2_@l-arginine-Cu(I) | 25 | 87 |


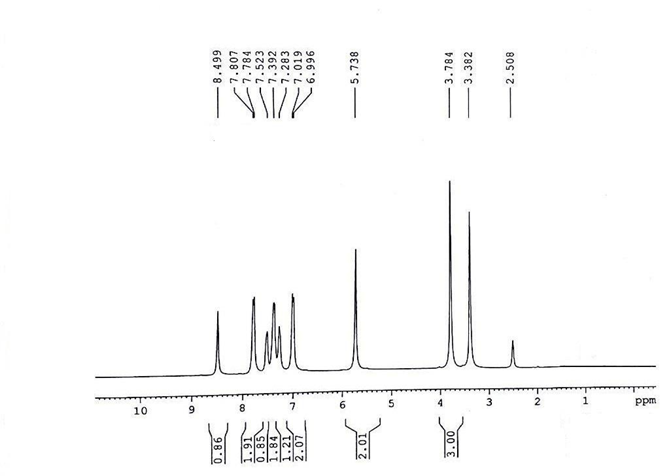


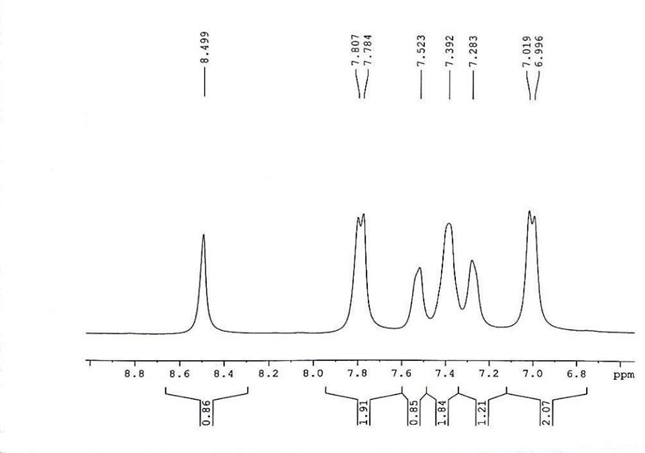


**Figure S 8.** ^1^HNMR spectra of the product 4h and expanded ^1^HNMR spectra of aromatic region.


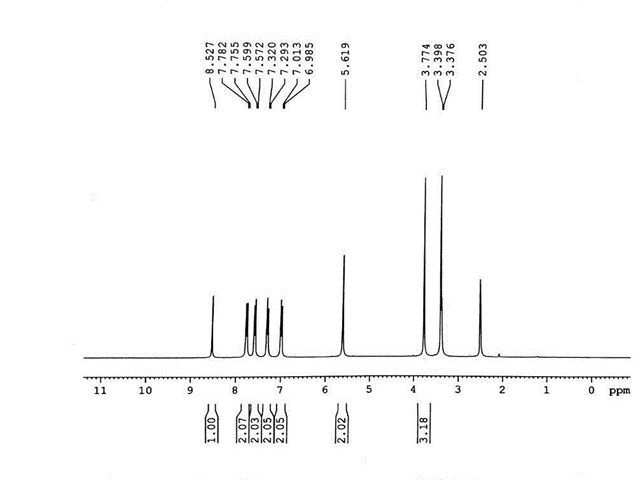

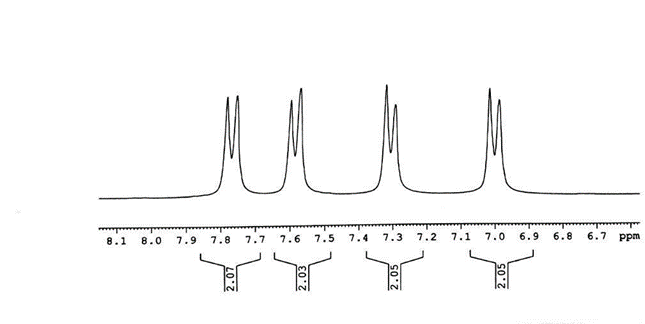


**Figure S 9.** ^1^HNMR spectra of the product 4i and expanded ^1^HNMR spectra of aromatic region.
